# The role of intraabdominal drain placement in minimal-invasive right hemicolectomy with complete mesocolic excision – a propensity score matched single center analysis

**DOI:** 10.1007/s00384-025-04948-0

**Published:** 2025-07-12

**Authors:** Maximilian Brunner, Katja Bondartschuk, Axel Denz, Georg F. Weber, Robert Grützmann, Christian Krautz

**Affiliations:** Department of General and Visceral Surgery, Friedrich-Alexander-University, Krankenhausstraße 12, Erlangen, Germany

**Keywords:** Right hemicolectomy, Complete mesocolic excision, Drain placement, Postoperative morbidity, Enhanced recovery after surgery, Optimized perioperative management

## Abstract

**Background:**

The role of intraabdominal drains in minimally invasive right hemicolectomy with complete mesocolic excision (CME) remains controversial. This study evaluates the impact of drain placement on perioperative outcomes using a propensity score-matched analysis in a single-center cohort.

**Methods:**

Data from 185 patients who underwent minimally invasive right hemicolectomy with complete mesocolic excision and central vascular ligation at our institution from 2016 to November 2024 were analyzed, including 62 without drains and 123 with drains. After propensity score matching, 50 patients from each group were compared. Postoperative outcomes were assessed between the groups and multivariate analysis was performed to identify risk factors for postoperative morbidity.

**Results:**

Postoperative complications, including morbidity (18% vs. 24%, p = 0.624), anastomotic leakage (2% vs. 2%, p = 1.000), surgical site infections (4% vs. 4%, p = 1.000) and re-surgery rate (2% vs. 6%, p = 0.617), did not differ significantly. However, the drain group showed delayed recovery milestones: longer time to first stool (2.1 vs. 2.7 days, p = 0.041), completion of meal plan (4.0 vs. 4.3 days, p = 0.038) and prolonged hospital stay (7 vs. 8 days, p = 0.045). Enhanced recovery rates were higher in the no-drain group (48% vs. 28%; p = 0.039). Multivariate analysis identified preoperative hemoglobin level ≤ 13 g/dl as a significant risk factor of postoperative complications (OR 9.8; 95% CI 2.0–48.7; p = 0.005), while drain placement was not significantly associated (p = 0.341).

**Conclusion:**

In minimally invasive right hemicolectomy with CME, routine drain placement does not reduce postoperative morbidity but may delay recovery milestones and prolong hospital stay. These findings suggest that selective rather than routine use of drains should be considered.

## Introduction

Minimally invasive techniques have revolutionized the field of oncological colorectal surgery, offering significant benefits in terms of reduced postoperative pain, shorter hospital stays and quicker recovery times [1–3]. Right hemicolectomy, a common procedure for patients with right-sided colon cancer and other conditions, has increasingly been performed using these minimally invasive approaches [4, 5]. Despite advancements in standardizing the procedure and the increasing adoption of optimized perioperative management protocols (e.g., enhanced recovery after surgery or fast-track), certain practices, such as the placement of intra-abdominal drains, remain debated [6].

The routine use of drains in colorectal surgery, including right hemicolectomy, is intended to indicate complications such as intraabdominal hemorrhage or anastomotic leaks and to prevent intra-abdominal fluid collections or abscess formation. However, their necessity is controversial [6–12]. A high-quality Cochrane review from 2004 (updated in 2016), which included three randomised controlled trials (RCTs) with a total of 908 patients who had undergone open colorectal resections, found no differences in the rates of anastomotic leakage, wound infection, re-intervention or mortality between the intervention (no drain) and control (drain) groups [8]. In addition, some studies suggest that drains potentially hinder recovery after surgery by adding to patient discomfort. Fast-track or enhanced recovery after surgery (ERAS) protocols aimed at reducing surgical stress and promoting early recovery, conflict with traditional practices like routine drain placement, underscoring the need to reassess their role in minimally invasive surgeries [13–16].

This study aims to contribute to the evidence by comparing the outcomes and recovery of patients undergoing minimally invasive right hemicolectomy with complete mesocolic excision (CME) and central vascular ligation (CVL) with and without the use of intra-abdominal drains. To ensure the robustness of the findings, propensity score matching was employed to adjust for any potential confounding variables.

## Materials and methods

We retrospectively reviewed data from 185 consecutive patients who underwent minimally invasive oncological right hemicolectomy between 2016 and November 2024 at the University Hospital Erlangen (total cohort). Non-oncological resections and open surgical procedures were excluded. The primary indication for surgery was malignancy, with a small number of cases involving premalignant lesions necessitating oncological resection. Patients were stratified according to the use an intraabdominal drain in two groups: drain vs. no drain.

To minimize confounding factors, propensity score matching was applied, considering the following parameters: age, sex, body mass index (BMI), ASA (American Society of Anesthesiologists) status, operative duration, surgical approach (laparoscopic vs. robotic), blood loss and kind of anastomosis (intracorporeal vs. extracorporeal). Nearest neighbor matching was conducted in a 1:1 ratio without replacement, using a caliper width of 0.2 standard deviations (SD) [17]. After matching, 50 patients in each group (drain vs. no drain) were analyzed for differences in postoperative outcomes (Matched cohort).

Data collected included patient demographics, comorbidities, preoperative parameters, intraoperative findings and postoperative outcome. Morbidity was classified using the Clavien-Dindo Classification, with any deviation from the normal postoperative course considered a complication [18]. Anastomotic leakage was defined according to the criteria of the International Study Group of Rectal Cancer and diagnosed based on a combination of clinical signs (e.g. abdominal pain, fever, elevated inflammatory markers), radiological imaging (primarily contrast-enhanced CT scans) and/or intraoperative confirmation during reoperation [19]. Postoperative ileus was defined as the absence of bowel function accompanied by abdominal distension, nausea or vomiting and the requirement for nasogastric decompression beyond postoperative day 3. Postoperative pain was evaluated using the Visual Analog Scale (VAS), while surgical site infections (SSIs) were categorized based on CDC (Centers for Disease Control and Prevention) guidelines for superficial incisional SSIs [20]. Additionally, a composite"enhanced recovery"parameter was developed, defined by the following criteria: first bowel movement by postoperative day (POD) 2, complete meal intake by POD 4, and discharge by POD 6.

The main focus was to compare the short-term postoperative outcome, including postoperative complications and postoperative recovery times, between the drain groups.

The study was conducted in accordance with the STROBE reporting guidelines [21].

### Surgical techniques

All patients underwent preoperative mechanical bowel cleansing using Clean Prep. Surgeries were performed under general anesthesia adhering to a standardized technique for minimal invasive hemicolectomy with CME and CVL [22]. At the beginning of the study period, the placement of a drain was our standard practice, while towards the end of the study period, omission of the drain became standard. However, the decision to place a drain was always at the discretion of the surgeon. There were no clearly defined criteria guiding this decision. In most cases, the drain was placed in the pelvis.

### Statistical analysis

Data were analyzed using SPSS software (version 24.0). Continuous and ordinal data were compared using the Student’s t-test or Mann–Whitney U test, while categorical variables were assessed using the Chi-square test. A p-value of < 0.05 was considered statistically significant. Multivariate analysis including parameters with a p < 0.1 in univariate analysis was performed to identify factors that may impact postoperative morbidity.

## Results

### Demographics

The total cohort contains of 185 patients (median age: 65 years, 51% female) with 62 patients receiving no intraabdominal drain and 123 patients receiving an intraabdominal drain during minimally invasive right hemicolectomy with CME and CVL. After applying propensity score matching, 50 patients in each group (drain vs. no drain) were analyzed.

Regarding the demographic data, the total cohort differed significantly between the two groups regarding sex (63% vs. 45% female, p = 0.030), BMI (24.2 vs. 25.9 kg/m^2^, p = 0.023), presence of diabetes (5 vs. 17%, p = 0.020) and preoperative creatinine (0.8 vs. 0.9 mg/dl, p = 0.013) (Table 1). In the matched group no differences in patients’ demographics were still evident (Table 1).

**Table 1:** Patient demographics

	**Total cohort**			**Matched cohort**		
	**No drain** **(n = 62)**	**Drain** **(n = 123)**	**p-value**	**No drain** **(n = 50)**	**Drain** **(n = 50)**	**p-value**
Mean age (years), median (IQR)	64 (19)	65 (23)	0.718	66 (18)	65 (26)	0.687
Sex, n (%) Male Female	23 (37) 39 (63)	67 (55) 56 (45)	**0.030**	21 (42) 29 (58)	25 (50) 25 (50)	0.547
BMI (kg/m^2^), median (IQR)	24.2 (7.2)	25.9 (6.0)	**0.023**	25.3 (6.4)	24.1 (5.8)	0.376
ASA, n (%) 1 2 3 4	4 (7) 48 (77) 10 (16) 0 (0)	6 (5) 79 (64) 35 (29) 3 (2)	0.145	3 (6) 38 (76) 9 (18) 0 (0)	3 (6) 39 (78) 8 (16) 0 (0)	1.000
Immunosuppression/steroid therapy, n (%)	4 (7)	10 (8)	0.777	3 (6)	6 (12)	0.487
Comorbidities, n (%) Arterial hypertension Cardiac disease Diabetes mellitus	29 (47) 8 (13) 3 (5)	60 (49) 27 (22) 21 (17)	0.876 0.166 **0.020**	24 (48) 5 (10) 3 (6)	22 (44) 10 (20) 7 (14)	0.841 0.262 0.318
Prior abdominal surgery, n (%)	19 (31)	48 (39)	0.331	15 (30)	17 (34)	0.830
Neoadjuvant chemotherapy, n (%)	3 (5)	1 (1)	0.110	0 (0)	3 (6)	0.243
Tumor localization, n (%) Terminal ileum/appendix/cecum Ascending colon Right flexure/right transverse colon	29 (47) 25 (40) 8 (13)	58 (47) 54 (44) 11 (9)	0.691	24 (48) 18 (36) 8 (16)	22 (44) 23 (46) 5 (10)	0.528
Preoperative blood results, median (IQR) White blood cell count (× 10^9^/l) Hemoglobin (g/dl) CRP (mg/l) Creatinine (mg/dl) Albumin (g/l) (n = 78)* CEA (ng/ml) (n = 94)*	6.9 (2.7) 12.9 (2.0) 2 (5) 0.8 (0.2) 42.3 (5.0) 2.1 (3.0)	6.4 (2.9) 12.3 (3.6) 3 (6) 0.9 (0.2) 42.2 (5.0) 1.8 (5.0)	0.422 0.327 0.565 **0.013** 0.722 0.649	7.0 (2.6) 12.9 (2.0) 3 (5) 0.8 (0.2) 42.7 (5.0) 2.3 (3.0)	6.2 (3.0) 12.3 (3.4) 3 (5) 0.8 (0.3) 41.8 (6.0) 1.5 (6.0)	0.529 0.280 0.693 0.511 0.184 0.564

IQR = Interquartile range; BMI = Body Mass Index; ASA = American Society of Anesthesiologists; CRP = C-reactive protein; CEA = Carcinoembryonic antigen; * not always determined

### Surgical parameters

The primary indication for surgery was a malignant lesion in the right colon (79%), with premalignant lesions accounting for the remaining cases (21%). The predominant surgical approach was laparoscopic (85%). Intra- and extracorporeal anastomoses were used with similar frequency. During surgery, 66% of patients received a drain placement. The median operative time and intraoperative blood loss were 210 min and 50 ml, respectively.

Regarding surgical parameters, the use of an intra-abdominal drain was significantly associated with a longer operative time (193 vs. 215 min, p < 0.001) in the total cohort. However, in the matched group, there were no differences in surgical parameters (Table 2).

**Table 2:** Surgical and pathological details

	**Total cohort**			**Matched cohort**		
	**No drain** **(n = 62)**	**Drain** **(n = 123)**	**p-value**	**No drain** **(n = 50)**	**Drain** **(n = 50)**	**p-value**
Indication for surgery, n (%) Malignant lesion Premalignant lesion	50 (81) 12 (19)	97 (79) 26 (21)	0.849	42 (84) 8 (16)	35 (70) 15 (30)	0.153
Surgical approach, n (%) Laparoscopic Robotic	49 (79) 13 (21)	108 (88) 15 (12)	0.131	42 (84) 8 (16)	43 (86) 7 (14)	1.000
Kind of anastomosis, n (%) Intracorporeal Extracorporeal	31 (50) 31 (50)	57 (46) 66 (54)	0.644	26 (52) 24 (48)	21 (42) 29 (58)	0.423
Operative time (min.), median (IQR)	193 (56)	215 (68)	** < 0.001**	210 (56)	208 (56)	0.591
Intraoperative blood loss (ml), median (IQR)	50 (70)	75 (50)	0.063	50 (55)	50 (50)	0.864
T category (n = 145/75)*, n (%) pT0/1 pT2/3	21 (44) 27 (56)	43 (44) 54 (56)	1.000	18 (45) 22 (55)	17 (49) 18 (51)	0.819
N category (n = 145/75)*, n (%) pN0 pN +	35 (73) 13 (27)	73 (75) 24 (25)	0.840	30 (75) 10 (25)	30 (86) 5 (14)	0.386
Resection margin, n (%) R0 R1	60 (98) 1 (2)	122 (99) 1 (1)	1.000	51 (98) 1 (2)	50 (100) 0 (0)	0.485

IQR = Interquartile range; * only for patients with malignant lesions

### Postoperative outcome

Morbidity was observed in 25% of all patients, with the majority (64%) classified as minor complications. These included anastomotic leakage in 2%, postoperative ileus or subileus in 2% and surgical site infections in 3% of cases. Re-surgery was required in 4% of patients. We found no morbidity attributable to the presence of a drain in our cohort.

**Fig. 1:**
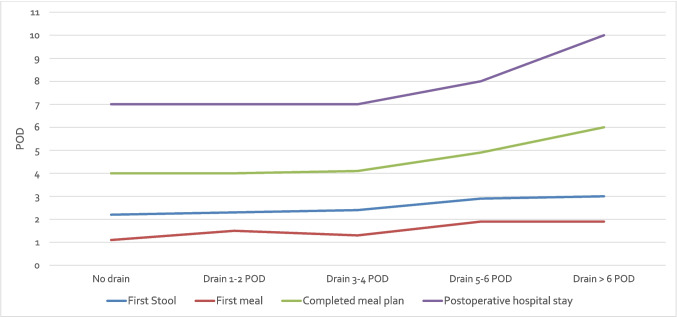
Postoperative recovery parameters stratified by duration of intraabdominal drain placement in the total cohort (first stool (p = 0.018), first meal (p = 0.004), completed meal plan (p = 0.002) and postoperative hospital stay (p < 0.001)). POD = Postoperative days

In the total cohort, postoperative complications were more frequent in the drain group (30%) compared to the no-drain group (16%) (p = 0.049). However, no significant differences were found between the groups in terms of complication severity (p = 0.406), the occurrence of anastomotic leakage (p = 1.000), postoperative ileus (p = 1.000), surgical site infections (p = 1.000) or the need for re-surgery (p = 0.271). Postoperative pain levels were comparable between the two groups, with no significant differences in pain scores at rest or under stress on postoperative days (POD) 1 to 5. Recovery parameters were significantly better in the no-drain group: The median day of first bowel movement occurred earlier in the no-drain group (POD 2.2 vs. 2.7, p = 0.023), as did the median day of first meal (POD 1.1 vs. 1.6, p < 0.001) and full meal intake (POD 4.0 vs. 4.8, p = 0.003). Additionally, hospital stay was significantly shorter in the no-drain group (7 vs. 8 days, p = 0.002). The composite"enhanced recovery"parameter was met by 45% of patients in the no-drain group, compared to 24% in the drain group (p = 0.004) (Table 3). Recovery parameters (first stool, first meal, completed meal plan and postoperative hospital stay) significantly worsened with increasing duration of intraabdominal drain placement (p = 0.018, p = 0.004, p = 0.002 and p < 0.001, respectively) (Fig. 1).

**Table 3:** Outcome parameters

	**Total cohort**			**Matched cohort**		
	**No drain** **(n = 62)**	**Drain** **(n = 123)**	**p-value**	**No drain** **(n = 50)**	**Drain** **(n = 50)**	**p-value**
Morbidity, n (%)	10 (16)	37 (30)	**0.049**	9 (18)	12 (24)	0.624
Clavien-Dindo, n (%) I II III IV V	2 (3) 6 (10) 1 (2) 1 (2) 0 (0)	7 (6) 14 (11) 10 (8) 4 (3) 2 (2)	0.406	1 (2) 6 (12) 1 (2) 1 (2) 0 (0)	3 (6) 5 (10) 2 (4) 2 (4) 0 (0)	0.835
Anastomotic leakage, n (%)	1 (2)	3 (2)	1.000	1 (2)	1 (2)	1.000
Postoperative ileus/subileus, n (%)	1 (2)	2 (2)	1.000	0 (0)	0 (0)	1.000
Surgical site infection, n (%)	2 (3)	4 (3)	1.000	2 (4)	2 (4)	1.000
Re-surgery, n (%)	1 (2)	7 (6)	0.271	1 (2)	3 (6)	0.617
First stool (POD), mean (SD)	2.2 (0.9)	2.7 (1.2)	**0.023**	2.1 (0.9)	2.7 (1.3)	**0.041**
First meal (POD), mean (SD)	1.1 (0.4)	1.6 (1.6)	** < 0.001**	1.1 (0.4)	1.4 (0.7)	**0.043**
Completed meal plan (POD), mean (SD)	4.0 (1.9)	4.8 (2.3)	**0.003**	4.0 (1.9)	4.3 (1.4)	**0.038**
Drain removal (POD), median (IQR)	-	5 (3)	**-**	-	4 (3)	**-**
Postoperative pain (VAS) at rest, mean (SD) POD 1 POD 2 POD 3 POD 4 POD 5	1.7 (2.0) 1.3 (1.6) 0.9 (1.4) 0.5 (0.9) 0.4 (0.9)	1.9 (1.8) 1.2 (1.6) 0.9 (1.3) 0.9 (1.6) 0.9 (1.7)	0.356 0.603 0.787 0.338 0.209	1.8 (2.0) 1.4 (1.7) 0.6 (1.0) 0.5 (1.0) 0.4 (0.9)	1.6 (1.5) 1.0 (1.3) 0.8 (1.4) 0.9 (1.7) 0.8 (1.9)	0.781 0.407 0.646 0.241 0.595
Postoperative pain (VAS) under stress, mean (SD) POD 1 POD 2 POD 3 POD 4 POD 5	2.7 (2.2) 2.4 (2.1) 1.5 (1.7) 1.2 (1.4) 0.8 (1.4)	3.1 (2.2) 2.3 (2.0) 1.6 (1.7) 1.6 (2.0) 1.5 (2.0)	0.296 0.959 0.724 0.577 0.061	2.7 (2.3) 2.4 (2.2) 1.2 (1.4) 1.1 (1.3) 0.8 (1.4)	3.1 (2.0) 2.2 (1.7) 1.7 (1.8) 1.7 (2.1) 1.4 (1.9)	0.327 0.961 0.264 0.452 0.144
Length of postoperative hospital stay (days), median (IQR)	7 (2)	8 (3)	**0.002**	7 (2)	8 (3)	**0.045**
Enhanced recovery*	28 (45)	29 (24)	**0.004**	24 (48)	14 (28)	**0.039**

POD = Postoperative day; SD = Standard deviation; IQR = Interquartile range. * defined as first stool at least on POD 2, complete meal plan at least on POD 4 and discharge at least on POD 6

Among the four patients who developed anastomotic leakage, three had received a drain and one had not. In two of the three patients with a drain (66%), the leakage was initially detected based on abnormal drain output on postoperative days 3 and 5, respectively. In the remaining patient (33%), anastomotic leakage was suspected due to elevated inflammatory markers despite unremarkable drain output, leading to re-laparoscopy on postoperative day 3. In the patient without a drain, diagnosis was based on clinical findings on postoperative day 4.

**Fig. 2:**
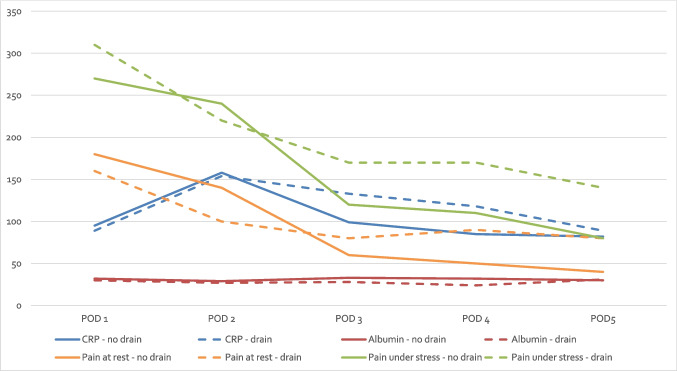
Postoperative course of CRP (in mg/l) and albumin (g/l × 10) levels as well as VAS scores (× 100) for pain at rest and under stress (each p > 0.05). POD = Postoperative days; CRP = C-reactive protein

In the matched cohort, postoperative morbidity (p = 0.624), severity of complications (p = 0.835) and the occurrence of anastomotic leakage (p = 1.000), postoperative ileus (p = 1.000), surgical site infections (p = 1.000) and re-surgery (p = 0.617) showed no significant differences between the groups. Postoperative pain scores (VAS) as well as postoperative CRP- and albumin-levels were similar between the drain and no-drain groups on POD 1–5 (each p > 0.05) (Fig. 2). Recovery parameters exhibited marked superiority in the no-drain group within the matched cohort: The median day of first bowel movement occurred earlier (POD 2.1 vs. 2.7, p = 0.041) and full meal intake was achieved sooner (POD 4.0 vs. 4.3, p = 0.038). Hospital stay was still significantly shorter for the no-drain group (7 vs. 8 days, p = 0.045). Additionally, 48% of patients in the no-drain group met the enhanced recovery criteria, compared to 28% in the drain group (p = 0.039) (Table 3).

### Multivariate analysis for postoperative morbidity

Multivariate analysis of the matched cohort revealed that preoperative hemoglobin level ≤ 13 g/dl was a significant risk factor for postoperative morbidity (OR 9.8; 95% CI 2.0–48.7; p = 0.005). However, the presence of an intraabdominal drain did not significantly impact the risk of postoperative complications (OR 1.7, 95% CI 0.6–5.2, p = 0.341) (Table 4).

**Table 4:** Multivariate risk analysis for occurrence of postoperative complications in a matched patient cohort

	**Matched cohort**			
	**Univariate**	**Multivariate**		
		**OR**	**95%-CI**	**p**
Mean age (≤ 65 vs. > 65 years)	0.629	-	-	-
Sex (Male vs. Female)	0.809	-	-	-
BMI (≤ 25 vs. > 25 kg/m^2^)	0.629	-	-	-
ASA (1/2 vs. 3/4)	0.514	-	-	-
Immunosuppression (No vs. Yes)	0.089	3.6	0.6–21.6	0.168
Arterial hypertension (No vs. Yes)	0.139	-	-	-
Diabetes mellitus (No vs. Yes)	1.000	-	-	-
Cardiac disease (No vs. Yes)	0.299	-	-	-
Prior abdominal surgery (No vs. Yes)	0.600	-	-	-
Neoadjuvant chemotherapy (No vs. Yes)	1.000	-	-	-
Tumor localization (Terminal ileum/appendix/cecum vs. Ascending colon vs. Right flexure/right transverse colon)	0.524	-	-	-
White blood cell count (≤ 11.5 vs. > 11.5 × 10^9^/l)	1.000	-	-	-
Hemoglobin (≤ 13 vs. > 13 g/dl)	**0.002**	**0.1**	**0.0–0.5**	**0.005**
CRP (≤ 5 vs > 5 mg/l)	0.058	1.8	0.6–6.0	0.308
Creatinine (≤ 1 vs. > 1 mg/dl)	0.731	-	-	-
Indication for surgery (Malignant lesion vs. Premalignant lesion)	0.246	-	-	-
Surgical approach (Laparoscopic vs. Robotic)	1.000	-	-	-
Kind of anastomosis (Intracorporeal vs. Extracorporeal)	0.462	-	-	-
Operative time (≤ 210 vs. > 210 min.)	1.000	-	-	-
Intraoperative blood loss (≤ 100 vs. > 100 ml)	0.169	-	-	-
Drain placement (No vs. Yes)	0.624	1.7	0.6–5.2	0.341

BMI = Body Mass Index; ASA = American Society of Anesthesiologists; CRP = C-reactive protein

## Discussion

Despite the consensus among most meta-analyses and reviews on intra-abdominal drains in colorectal surgery that their placement does not prevent the most feared complication – anastomotic leakage – nor other postoperative complications, drain placement remains a common practice among many surgeons [8–11]. This issue is becoming increasingly significant as the deliberate avoidance of drain insertion is an integral component of fast-track and enhanced recovery after surgery (ERAS) protocols, which are meticulously designed to facilitate recovery by minimising interventions that could potentially compromise patient homeostasis [13–16].

It is imperative to accentuate that a substantial proportion of existing evidence on this subject is derived from heterogeneous cohorts of colorectal surgeries, frequently encompassing patients with rectal anastomoses or data predominantly collected prior to the implementation of minimally invasive techniques and CME surgery [7–12]. For this reason, we deliberately chose a homogeneous study population of patients undergoing minimally invasive right hemicolectomy with CME and CVL. To our knowledge, only one prior study has investigated this specific patient population, which underscores the need for further research [6].

Our study demonstrates that the placement of intra-abdominal drains was not associated with a reduced incidence of postoperative complications. The higher complication rate observed in the drain group can be attributed to the fact that more complex cases were preferentially managed with drains, as the decision to place a drain was made intraoperatively by the surgeon. This assumption is supported by our finding that, after propensity score matching for patient characteristics and surgical approach, the difference in complication rates between the groups was no longer observed. These findings align with the study by Solaini et al., which analyzed outcomes in 653 patients undergoing right hemicolectomy and reported similar conclusions [6].

The placement of abdominal drains has traditionally been employed to identify potential intra-abdominal hemorrhage, anastomotic leaks and reduce the risk of pelvic or abdominal sepsis by allowing the drainage of fecal or purulent discharge. Additionally, the use of intra-abdominal drains is thought to prevent postoperative fluid accumulation at the anastomotic site, thereby potentially lowering the risk of infection. Although some randomized controlled trials have suggested a potential benefit of abdominal drains in reducing anastomotic leakage rates, our study did not demonstrate a significant reduction in anastomotic leaks or other severe complications classified as Clavien-Dindo grade III or higher [23, 24]. These findings align with the majority of published studies and meta-analyses on this topic [6–12]. Although two out of three patients with anastomotic leakage and a drain in place were initially diagnosed based on abnormal drain output, suggesting that drainage may have some value for early detection, the low incidence of anastomotic leaks in our cohort precludes drawing definitive conclusions regarding the potential benefit of drains for early leak diagnosis.

Similarly, no significant differences were observed between the drain and no-drain groups in terms of other specific complications, such as postoperative ileus or surgical site infections. This is noteworthy, as some studies have identified drain placement as a potential risk factor for infections [7, 25]. Our findings are consistent with the conclusions of the comparable study by Solaini et al. [6], further supporting the limited utility of routine drain placement in minimally invasive oncological right hemicolectomy.

Despite the lack of significant differences in postoperative complications in the matched cohort, patients in the no-drain group exhibited significantly better recovery outcomes. This included earlier restoration of bowel function, faster progression to full oral intake and shorter hospital stays. The composite"enhanced recovery"parameter further underscored these findings, with nearly half (48%) of the no-drain group meeting the enhanced recovery criteria, compared to only 28% in the drain group (p = 0.039). These results highlight the potential benefits of omitting drains as part of Fast-track or ERAS protocols, which focus on optimizing recovery and reducing hospital stays [13–16, 26].

The principles of optimized perioperative management emphasize minimizing surgical stress and avoiding unnecessary interventions that could delay recovery [13–16]. Our study contributes to the growing body of evidence supporting the omission of drains as a component of such pathways in colorectal surgery. The earlier return of bowel function, faster oral intake progression and shorter hospital stays observed in the no-drain group are key indicators of successful Fast-track or ERAS protocol implementation. By contrast, the use of drains may interfere with patient mobilization and comfort, potentially hindering recovery.

Our data suggest that not only the indication for drain placement, but also the duration of drainage should be carefully considered in patients with drains, as prolonged drainage may impair postoperative recovery. In a subgroup analysis, patients with drains in place for ≥ 5 days showed particularly impaired recovery (Fig. 1). However, as these results are based on the unmatched cohort, a relevant selection bias must be considered and causality cannot be established, as prolonged drainage may also reflect more complex postoperative courses.

Multivariate analysis in our study identified a preoperative hemoglobin level ≤ 13 g/dl as a significant predictor of postoperative morbidity, highlighting the critical importance of prehabilitation and meticulous intraoperative hemostasis and supporting previous studies [27, 28]. However, the use of an intra-abdominal drain was not found to be an independent risk factor for complications. Instead, the decision to place a drain is likely influenced by the surgeon's subjective intuition regarding anastomotic healing, which may introduce bias into observational studies.

The present findings are consistent with a recent meta-analysis questioning the routine use of drains in minimally invasive colorectal surgery [11]. While drains may provide benefits in certain high-risk cases, such as those with significant intraoperative contamination or technically challenging anastomoses, the evidence supporting their routine use in right hemicolectomy with CME is inconclusive. Our study supports this perspective by showing that omitting drains does not appear to increase the risk of complications, even in a matched cohort.

Nonetheless, several limitations of our study must be acknowledged. First, the retrospective design introduces the potential for selection bias, even with the use of propensity score matching. Second, the relatively small sample size in the matched cohort may limit the statistical power to detect differences in rare complications, such as anastomotic leakage. Third, the decision to place a drain was left to the discretion of the operating surgeon, potentially introducing selection bias based on intraoperative findings. Surgeons may have been more inclined to place a drain in cases they perceived as more complex or with a higher intraoperative risk, for example due to technical difficulties, intraoperative bleeding, or questionable anastomotic integrity. As a result, the drain group may have included a disproportionate number of higher-risk patients, which could have influenced postoperative outcomes independently of the drain itself. Although propensity score matching was applied to adjust for known confounders, unmeasured factors related to intraoperative decision-making may still have led to residual confounding. This limitation should be considered when interpreting the results. Finally, the study was conducted at a single high-volume tertiary center, which may limit the generalizability of the findings to other institutions with differing surgical expertise and patient populations.

## Conclusion

The present propensity score-matched study supports the growing consensus that routine drain placement may not be necessary in minimally invasive right hemicolectomy with CME and CVL. In fact, the omission of drains was associated with faster recovery, shorter hospital stays and comparable complication rates, aligning with the principles of enhanced recovery. However, the findings should be interpreted with caution due to the study’s limitations and the small absolute differences observed in recovery parameters.

## Data Availability

No datasets were generated or analysed during the current study.

## Declarations

### Statement on the welfare of animals

This article does not contain any studies with animals performed by any of the authors.
